# Primary healthcare rehabilitation users’ views on activity limitations and participation in South Africa

**DOI:** 10.4102/ajod.v13i0.1391

**Published:** 2024-10-21

**Authors:** Lebogang J. Maseko, Fasloen Adams, Hellen Myezwa

**Affiliations:** 1 Department of Occupational Therapy, School of Therapeutic Sciences, Faculty of Health Sciences, University of the Witwatersrand, Johannesburg, South Africa; 2 Department of Health and Rehabilitation Sciences, Division of Occupational Therapy, Faculty of Medicine and Health Sciences, Stellenbosch University, Stellenbosch, South Africa; 3 Department of Physiotherapy, School of Therapeutic Sciences, Faculty of Health Sciences, University of the Witwatersrand, Johannesburg, South Africa

**Keywords:** rehabilitation service users, person-centred approach, integrated rehabilitation services, rehabilitation outcomes, Primary Healthcare, functional limitations, occupational therapy, physiotherapy, speech and language therapy, audiology

## Abstract

**Background:**

Increasing functional limitations and disabilities have raised the need for comprehensive rehabilitation services at the primary healthcare (PHC) level, particularly in low- and middle-income countries. To support the integration of these services into PHC in South Africa, assessing outcomes from the service users’ perspectives is essential.

**Objectives:**

This study examined service users’ views on their PHC rehabilitation outcomes in a Metropolitan District of Gauteng, South Africa. The aim was to understand perceived changes in activity limitations and participation restrictions following the rehabilitation intervention.

**Method:**

A quantitative survey design, including self-rating measurements and structured interviews, was employed. Thirty-eight rehabilitation service users from eight clinics and community health centres were purposively sampled. Participants rated their pre- and post-rehabilitation levels of difficulty in activity limitations and participation restrictions, with open-ended questions providing additional insights. Data analysis used descriptive statistics, quantitative content analysis, and non-parametric tests.

**Results:**

Significant improvements in mobility, self-perception, and quality of life were reported by both adult and child service users. Caregivers of child service users also noted positive experiences (*p* = 0.019) in community, social, and civic life.

**Conclusion:**

This study highlights the perceived positive changes experienced by PHC rehabilitation service users in addressing functional limitations and disabilities. It underscores the effectiveness of integrated rehabilitation service delivery in improving user outcomes.

**Contribution:**

The findings offer valuable insights into how rehabilitation interventions enhance functional abilities, social participation, and overall well-being. By focusing on activity limitations and participation restrictions from service users’ perspectives, this study supports the priority of providing person-centred rehabilitation services at the PHC level.

## Introduction

The global increase in non-communicable diseases, neurological disorders, and traumatic injuries has led to a rise in functional limitations and disabilities, particularly in low and middle-income countries such as South Africa. Consequently, there is a growing demand for comprehensive rehabilitation services (Stucki et al. 2017; World Health Organization 2019) to address this need. The WHO and the United Nations Children’s Fund (2018) advocate for the provision of rehabilitation services at the primary healthcare (PHC) level for individuals of all ages using a whole-society approach to address the determinants of health, and by bringing services closer to communities. However, despite the known importance of rehabilitation services, their integration at the PHC level has been hindered globally by a lack of recognition by policy makers and service planners (Joint Learning Network 2023).

One potential reason for this limited integration may be the lack of consideration of rehabilitation in health systems planning and an insufficient understanding of the benefits of rehabilitation outcomes on health, disability and quality of life (QoL) as defined by the WHO (2002). Contrary to the medical model that underpins most health systems and focuses primarily on body structures and functions, rehabilitation adopts a biopsychosocial spiritual approach to health as defined in the International Classification of Functioning, Disability and Health (ICF). This approach considers activity limitations, and participation restrictions as well as personal and environmental factors (Stucki & Höök 2016; WHO 2002). Rehabilitation services aim to enhance not only physical and mental functioning but also to support the purposeful engagement in daily life, including the domains of self-care, communication, social, leisure, work and/or education (WHO 2019).

Therefore, it is important to establish the outcomes of rehabilitation services, based on the ICF, determined by the individuals’ capacity or intrinsic ability to carry out actions in everyday activities across various domains in their physical, cultural, social, and economic environments (Huus et al. 2021; Stucki & Höök 2016). The outcomes are further quantified through measures of QoL and well-being (Donnelly et al. 2023). It is essential to provide evidence of these outcomes to enhance the understanding and promotion of the integration of rehabilitation services in PHC. To provide the best value to their users, health systems must first understand what is important to patients and carers, how to capture this information, and most importantly, how to use this information to improve the quality of care provided by healthcare systems (Cadel et al. 2022).

The South African health system shows apathy in integrating rehabilitation service delivery into their implementation of a re-engineered PHC, a strategy aimed at achieving universal health coverage in preparation for the National Health Insurance (NHI) (Louw et al. 2023). This is despite advocacy for the integration of rehabilitation into standard treatment guidelines for PHC being in the Framework and Strategy for Disability and Rehabilitation Services in South Africa (FSDRSA) (National Department of Health 2015). The proposed PHC multidisciplinary teams for the NHI primarily include nurses, community health workers, and doctors (Mash et al. 2020), with limited mention of rehabilitation services. Consequently, individuals with functional limitations and disabilities, or those at risk of disability could face limited access to rehabilitation services at the PHC level in the proposed NHI, a single healthcare system financed by the government (Morris et al. 2021).

To support the integration of rehabilitation into PHC, it is essential to provide evidence on the effectiveness of PHC rehabilitation services delivered by interprofessional teams comprising personnel from physiotherapy, occupational therapy, speech and language therapy, and audiology, among others (WHO 2019). Such evidence should support specific person-centred rehabilitation interventions delivered by rehabilitation professionals, which incorporate goal-setting to address impairments and compensate for disability, and provide environmental assessment, adaptation, as well as the prescription of specialised equipment (Brown et al. 2021). The evidence should emphasise the inclusion of client experiences in the evaluation of services aimed at restoring, reclaiming, or maintaining function (Meyer et al. 2014). Measuring patient experience provides a more comprehensive picture of healthcare quality and can highlight areas for improvement (Cadel et al. 2022). Patient experience encompasses the range of interactions patients have with the healthcare system, which may include several aspects of care delivery such as patient-provider communication and appointment wait times, to name a few. Additionally, emphasising community participation and health outcome evaluation are vital for providing clinical evidence, particularly in sub-Saharan Africa (Mash et al. 2020).

Literature on the effectiveness of PHC-level rehabilitation often focuses on specific diagnostic groups treated by a single rehabilitation discipline in high-income countries (Gell, Mroz & Patel 2017). Consequently, there is limited evidence regarding the effectiveness of such services on activity limitations and participation restrictions (Abdel-Malek, Rosenbaum & Gorter 2020). Notable improvements in social activity, work productivity, and reduced healthcare admissions have only been reported following interdisciplinary PHC rehabilitation interventions for chronic pain (Stein & Miclescu 2013).

In profession-specific publications in Sweden (Matérne et al. 2022) and Nigeria, physiotherapy interventions at the PHC level for elderly clients and stroke survivors show positive changes in mobility, health-related QoL, and reintegration into daily life (Olaleye, Hamzat & Owolabi 2014). Occupational therapy services in PHC show promising results in countries such as Sweden, the Netherlands, and the USA. Studies from these countries report improved occupational performance, return to work, and enhanced QoL for individuals with conditions such as depression, stress-related ill-health, dementia, frailty, and Parkinson’s disease (De Coninck et al. 2017; Eklund, Erlandsson & Wästberg 2015; Erlandsson 2013; Kjerstad & Tuntland 2016; Sturkenboom et al. 2014). Donnelly et al. (2023) also report improved participation in work, and functional and community mobility, including driving and engaging in social activities for adults of various ages. In the Netherlands, an online programme supporting adults with attention deficit hyperactivity disorder and autism has been successful in improving instrumental activities of daily living (Bolt et al. 2019a).

Research on outcomes in paediatric PHC rehabilitation services traditionally focuses on body functioning, but there is evidence of positive effects on activity limitations and participation restriction (Tveten et al. 2020). Although somewhat outdated, physiotherapy research indicates improvements in mobility and functional ability in children with cerebral palsy (Smetana 2012). Similarly, occupational therapy interventions for children with cerebral palsy show enhancements in occupational performance, self-care, play, and education, as assessed by caregivers (Imms et al. 2010; Kolit & Ekici 2023) Telehealth in paediatric PHC occupational therapy also demonstrates benefits in communication, school participation, and self-care for daily activities (Önal et al. 2021). However, no recent research reporting improvements in activity limitation and participation restrictions through speech and language therapy or audiology in PHC was found, indicative of the shortage of these services at the primary level. Despite the available literature, the evidence on the effectiveness of PHC-level rehabilitation services remains inadequate, particularly for functional outcomes related to activity limitations and participation restrictions based on the ICF framework. There is a significant gap in understanding the activity limitation and participation restriction changes experienced by PHC rehabilitation service users, particularly in low- and middle-income countries. This study aimed to address this gap by exploring the change in activity limitations and participation restriction outcomes of PHC rehabilitation services at eight PHC clinics funded by the provincial health department in a Metropolitan District in Gauteng, South Africa.

## Research methods and design

### Study design

The study used a quantitative survey design with a subjective, self-rating measurement accompanied by open-ended questions where data were collected during structured interviews. The inclusion of open-ended questions allowed participants to provide qualitative insights into the changes experienced in activity limitations and participation restrictions (Zhao & Kwok 1999).

### Study setting

The study was conducted in a Metropolitan Health District in Gauteng, South Africa, which comprises 125 PHC clinics and community health centres across seven regions (Massyn et al. 2020). The facilities provide services to over 100 000 people (Gauteng Province Co-operative Governance and Traditional Affairs 2021). Of these facilities, only nine offer rehabilitation services, including physiotherapy (22 staff members), occupational therapy (20 staff members), speech and language therapy (4 staff members), and audiology (2 staff members) (Jhb Metro Rehab 2023). At the time of the study, one of the nine clinics had diverted their rehabilitation services to another clinic, thus only eight facilities were available for the study.

### Study population and sampling strategy

Rehabilitation service users attending the clinics and community health centres in a Metropolitan District that offer rehabilitation services were recruited for the study. Purposive sampling was used at eight of nine provincial clinics and community health centres, where 80 rehabilitation service users, who met the inclusion criteria of receiving regular weekly or monthly services and being able to speak and understand English, were identified. Those who attended fewer than three rehabilitation sessions were excluded to ensure participants had an adequate experience of rehabilitation services when reporting outcomes (Maseko, Myezwa & Adams 2024). Considering a 10% margin of error, as recommended by Cochran’s formula for ordinal data, based on the 80 potential participants identified, 38 participants were included in the study (Oribhabor & Anyanwu 2020). The study collected data on the perceived outcomes for both adult and child service users from adults or their caregivers. These caregivers were either parents, legal guardians, or family members who provided the necessary care to adult service users. Adults or the caregivers of children, most of whom were too young for interviews, reported on the changes in activity limitations and participation restrictions before and after rehabilitation. Additionally, caregivers, with consent from adult service users with communication disorders, assisted or facilitated their participation in the interviews using strategies implemented in rehabilitation sessions. Data were collected over a 4-month period between March and June 2021. The eight research assistants, who were rehabilitation professionals, conducted the interviews. They were trained by the principal researcher on the data collection process and had no prior knowledge of the participants or the treatment they received.

### Data collection

A researcher-developed survey, administered by research assistants in a structured interview, was used to gather data. The survey included structured interview questions with open-ended prompts that aimed to provide additional details about participants’ experiences or their caregivers’ observations of changes from pre- to post-rehabilitation. Interviews were conducted in English and the interviewer and/or adult caregiver assisted participants when required. To ensure content validity, three rehabilitation professionals, one occupational therapist, one physiotherapist, and one speech and language therapist and audiologist with a dual qualification recognised as experts with over 30 years’ experience in public and private health systems in the field of rehabilitation, reviewed the survey questions. They assessed each question for relevance, clarity, ambiguity, and simplicity, rating them on a scale from 1 to 4 (Polit & Beck 2006). The final survey incorporated all relevant suggestions from their feedback.

The survey consisted of three sections. Section 1 included sociodemographic questions, medical history, and clinic visit information. Section 2 consisted of 12 basic self-rated items aligned with the ICF classification to assess difficulties in daily activities, mobility, employment, and schooling (Zhao & Kwok 1999). In section 2, participants were asked to rate their pre- and post-rehabilitation performance as *independent with full participation, independent with some help*, or *limited participation* verbally in reply to the questions. Answers provided in this section were confirmed in open-ended structured interview questions, and patients were also asked to describe their own or their child’s feelings about themselves and their QoL to gather more detailed information about changes experienced during rehabilitation. Section 3 of the survey included questions that asked participants to rate their self-perception, including their physical, mental, or social attributes, and quality of life (QoL) as ‘good,’ ‘neither good nor bad,’ or ‘bad’. This approach is based on the integrative theory of QoL, as suggested by Lindholt, Ventegodt, and Henneberg (2002), which considers both objective and subjective components.

Eight research assistants who were rehabilitation professionals employed at the clinics received training from the principal researcher in the survey administration and structured interview procedures. The rehabilitation professionals at the community health centres and clinics identified 80 eligible service users not previously treated by them, of which 38 were included in the study. Participants were provided with an information sheet, which was explained, and the information discussed with them where necessary. Written informed consent for study participation and audio recording was obtained from all adult participants and caregivers of the children. Interviews were conducted with adult service users, assisted by caregivers where necessary, or with caregivers of child service users, to discuss changes in their own or their child’s activity limitations and participation restrictions before and after rehabilitation. These interviews occurred in a quiet room within the clinic and were audio recorded using a tablet. Interview recordings were anonymised and transcribed verbatim.

### Data analysis

Demographic data, participants’ self-rated levels of independence in different domains of activity limitation and participation restrictions before and after rehabilitation, feelings about oneself, and QoL were analysed descriptively using frequencies based on the survey responses. Perceived changes in activity limitations and participation restrictions before and after rehabilitation were analysed using a chi-squared test (sig 0.05) as a non-parametric statistical method.

The qualitative data collected from the transcriptions of open-ended questions in responses to changes in activity limitations and participation restrictions, feelings about oneself, and QoL were coded and analysed using quantitative content analysis (Züll 2016). A categorisation scheme, using survey descriptors as coding categories, was developed. The scheme and categories underwent pilot testing by the two primary coders (the researcher and a colleague) and were revised until intercoder agreement was achieved (Züll 2016). Coding was completed using a Microsoft Excel spreadsheet. To confirm the quality of the coding, a third coder independently coded a sample of the responses (10% of all open-ended responses) for reliability. The findings were peer reviewed by two independent academic rehabilitation professionals with experience in PHC and research to establish agreement on all scores. To ensure validity of the participants’ or their caregivers’ ratings of performance in the domains of activity limitations and participation restrictions, the descriptive ratings were triangulated with the coded data to confirm consistency for each domain (Züll 2016). Data pertaining to adult service users and information provided by caregivers regarding their children, who were also service users, were analysed separately. This approach was employed to accommodate age-related differences in activities and participation, utilising distinct spreadsheets and codebooks for each group.

### Ethical considerations

Ethical clearance to conduct this study was obtained from the University of the Witwatersrand, Human Research Ethics Committee (Medical) (No. M190466). Participants, adult service users and caregivers gave written informed consent to participate in the study and be audio recorded following explanation and discussion about the study, and understood that participation was voluntary, and they could withdraw at any stage in the research process. Confidentiality during the data analysis, results and publication that would arise from the study was ensured through assigning participants a participant code and only the first author having access to the audio recordings of transcription.

## Results

### Sociodemographic information

Thirty-eight participants, including 21 adults and caregivers of 17 children, completed the survey (Table 1). Most adult participants were female (52.38%), and most children were boys (70.59%). The age distribution showed that most participants fell within middle childhood (3–7 years) and middle adulthood (41–60 years) age ranges. Nearly half of the participants receive a social grant. Additionally, 42.86% of adult participants had completed 12 years or more of education, but only one-third were employed and nearly a quarter were retired. Physiotherapy had the highest attendance rate among adults (62.50%), but the lowest among children (26.67%). Speech therapy had the lowest attendance among adults (12.50%), and audiology services were not routinely available at the Community Health Centres (CHCs) during the time of data collection, and were therefore referred to the higher level of care.

**TABLE 1:** Sociodemographic information of rehabilitation service users in primary healthcare facilities in a metropolitan district (*N* = 38).

Variable	Description	Adults (*n* = 21)		Children (*n* = 17)	
		*n*	%	*n*	%
Sex	Male	9	42.86	12	70.59
	Female	11	52.38	5	29.41
	Not recorded	1	4.76	0	0.00
Age at assessment (years)	< 3	N/A	N/A	3	17.65
	3–7	N/A	N/A	12	70.59
	11–20	1	4.76	N/A	N/A
	21–40	6	28.57	N/A	N/A
	41–60	10	47.62	N/A	N/A
	61–70	4	19.05	N/A	N/A
	Not recorded	0	0.00	2	11.76
Education level	None	3	14.29	9	52.94
	Pre-primary (Crèche–Gr R)	0	0.00	5	29.41
	Primary (Grade 1–7)	9	42.85	3	17.65
	Secondary (Grade 8–12)	6	28.57	0	0.00
	Post-secondary (Post Matric)	3	14.29	N/A	N/A
	Not recorded	9	0.00	0	0.00
Occupation status	Unemployed	9	42.86	N/A	N/A
	Employed	7	33.33	N/A	N/A
	Retired	5	23.81	N/A	N/A
Social grant	Yes	8	38.10	10	58.82
	No	13	61.90	7	41.18
	Not recorded	9	0.00	0	0.00
Specific rehabilitation services attended	Physiotherapy	20	62.50	8	26.67
	Occupational therapy	8	25.00	11	36.67
	Speech therapy	4	12.50	11	36.67

### Factors related to attendance at rehabilitation services

Most participants attended Clinic D in the Metropolitan District (Table 2). Based on the WHO impairments (body function) codes (WHO 2002), all the adult participants presented with neuromusculoskeletal and movement-related functions disorders (b7). Among the children, a small percentage had disorders in voice and speech function (b3) (5.9%), sensory function (b2) (11.8%), and mental function (b1) (17.7%). Most adult participants were referred for rehabilitation at the PHC level (38.1%), and most children were down referred from the tertiary level (53%). The majority of both adult and children service users attended rehabilitation sessions monthly (71.4% and 64.7%, respectively).

**TABLE 2:** Factors related to attending rehabilitation in primary healthcare facilities in a metropolitan district (*N* = 38).

Variable	Description	Adults (*n* = 21)		Children (*n* = 17)	
		*n*	%	*n*	%
CHC or Clinic	A	4	19.05	1	5.88
	B	2	9.52	1	5.88
	C	3	14.29	3	17.65
	D	1	4.76	6	35.29
	E	5	23.81	0	0.00
	F	4	19.05	0	0.00
	G	0	0.00	3	17.65
	H	2	9.52	3	17.65
Impairment- ICF body function and codes (WHO 2002)	Neuromusculoskeletal and movement-related functions (b7)	21	100.00	11	64.71
	Voice and speech functions (b3)	0	0.00	1	5.88
	Sensory functions and pain (b2)	0	0.00	2	11.76
	Mental functions (b1)	0	0.00	3	17.65
Referral source (level of care)	Quaternary	0	0.00	1	5.88
	Tertiary	7	33.33	9	52.95
	Secondary	4	19.05	1	5.88
	Primary/clinic	8	38.10	4	23.53
	Other	2	9.52	1	5.88
	Missing	0	0.00	1	5.88
Rehabilitation attendance	Weekly (1x a week)	6	28.67	6	35.39
	Monthly (1x a month)	15	71.43	11	64.71

ICF, International Classification of Functioning, Disability and Health; CHC, Community Health Centre.

### Change in activities and participation before and after rehabilitation in adults

For adults, the activity limitations and participation restriction areas assessed included self-care, domestic life, mobility, community, social and civic life, and major life areas related to work (Table 3).

**TABLE 3:** Change in activities and participation before and after rehabilitation for adult participants (*N* = 21).

Variable	Before		After		*P*
	*n*	%	*n*	%	
**Self-care (eating, washing, dressing)**					0.032*
No difficulty or independent	5	23.80	12	57.14	
Mild or moderate difficulty (May need some assistance, or slow and effortful)	10	47.61	6	28.57	
Severe or complete difficulty or not at all (Needs maximal assistance)	3	14.28	0	0.00	
Not specified	3	14.28	3	14.28	
**Domestic life (cooking, cleaning)**					0.652
No difficulty or independent	4	19.04	6	48.57	
Mild or moderate difficulty (May need some assistance, or slow and effortful)	1	4.76	-	-	
Severe or complete difficulty or not at all (Needs maximal assistance)	5	23.80	4	19.04	
Not specified	11	52.38	11	52.38	
**Mobility**					0.006*
No difficulty or independent in community	2	9.53	10	47.61	
Mild or moderate difficulty (inside and outside house)	9	42.85	5	23.60	
Severe or complete difficulty (Needs assistance)	6	48.57	1	4.76	
Not specified	4	19.04	5	23.80	
**Community, social and civic life (socialise, visiting, church)**					0.426
No difficulty (independent or often need assistance)	7	33.33	10	47.61	
Mild or moderate difficulty (sometimes with a familiar person only)	6	28.57	3	14.28	
Severe or complete difficulty (not at all or limited communication)	3	14.28	2	9.52	
Not specified	5	23.80	6	28.57	
**Major life areas (work)**					0.228
No difficulty or employed	1	4.76	5	23.80	
Able to work with difficulty or adapted work tasks	3	14.28	2	9.53	
Unable to work	8	38.09	3	14.28	
Retired or unemployed prior to disability	3	14.28	3	14.28	
Not specified	6	28.57	8	38.09	

Note:

* Significant ≤ 0.050.

Notable changes were observed in two domains elaborated in the following subsections.

#### Self-care (eating, washing, dressing)

Participants showed a significant improvement (*p* = 0.032) in their independence in eating, dressing, and bathing after rehabilitation. Some participants (28.57%) still required assistance with dressing and bathing, but overall, self-care abilities improved, as shown in the following comments:

‘After seeing [*therapist X*] … I was doing exercise that [*therapist X*] gave me … it … helps me … I was doing some of the things myself … not … helped by anyone.’ (P38, Male, 37 years)

‘A lot of change because … I can dress by myself, and I can bath by myself, and I can eat by myself.’ (P05, Male, 25 years)

#### Domestic life (cooking, cleaning)

Participants reported improved independence in household tasks, such as washing dishes and cleaning cupboards. Cooking and cleaning were not differentiated, except by one participant who is unable to walk but could independently wash dishes. One participant indicated that she could cook and clean but was unable to do laundry. One participant described his experience, saying:

‘I always bring dishes to wash them sitting down. Cleaning my cupboard, dishes inside, clean inside there … sitting down.’ (P30, Male, 64 years)

#### Mobility

A significant improvement (*p* = 0.006) in mobility was observed and participants reported increased ability to move around, walk, and perform activities. Six participants indicated that they were unable to move around without help before rehabilitation. One participant described her experience as follows:

‘And for me it was very difficult to move and to do any type of activity. And my brother helped me to move at home, and without his help it was very difficult.’ (P25, Female, 49 years)

Those using wheelchairs and crutches reported mild to moderate difficulty after rehabilitation, but one participant required assistance to push the wheelchair to move around. All the participants reported they had started to walk after intervention, even if only indoors. Some continued to rely on a wheelchair for long distances. They described their situations as follows:

‘I can’t walk. I just started now … my house is too little … but I walk by myself … in the yard … not in the street.’ (P32, Female, 67 years)

‘Now she can walk around … she walks with a walker in the house, in the yard … but street is too far … we fetch the wheelchair.’ (P14, Female, 56 years)

#### Community, social, and civic life

Most participants did not have trouble with socialising, visiting, or attending church after rehabilitation. One participant’s communication skills improved after intervention:

‘She’s much better … she can communicate with me … she talks to them [*family members*] … they obviously don’t understand her … family … she talks to them.’ (P24, Female, 55 years)

#### Major life areas (work)

A small percentage of participants were employed after rehabilitation and nearly 20% successfully returned to work. Only two participants were not ready to return to work. The participants described their situation as follows:

‘Not yet [*ready to go back to work*] … applied for a DG [*disability grant*].’ (P08, Male, 37 years)

‘I’m working … I do everything at work … I’m back … at where I worked [*before rehabilitation*] … everything is fine … I’m getting my own income.’ (P29, Female, 41 years)

### Feelings about oneself (self-perception) and quality of life in adults

Participants reported a marked improvement in self-perception (47.61%) and QoL after rehabilitation. Many participants developed a more positive sense of self, and statistically significant positive changes were observed in their QoL (42.85%; Table 4).

**TABLE 4:** Changes in feelings about self and quality of life before and after rehabilitation for adult participants (*N* = 21).

Variable	Before		After		*P*
	*n*	%	*n*	%	
**Feelings about self**					0.014*
Good	4	19.04	10	47.61	
Neither good nor bad	8	38.09	2	9.53	
Bad	5	23.80	1	4.76	
Not specified	4	19.04	8	38.09	
**QoL**					0.017*
Good	2	9.53	9	42.85	
Neither good nor bad	8	38.09	7	33.33	
Bad	6	28.57	1	4.76	
Not specified	5	23.80	5	23.80	

Note:

* Significant ≤ 0.050.

QoL, quality of life.

The following quotes are examples of the participants’ experiences before rehabilitation:

‘I was feeling negative about myself because of the way I was … I thought … that’s the end of the story with me because … I couldn’t walk. I couldn’t do anything. I was crippled.’ (P07, Male, 38 years)

‘Poor … I couldn’t do anything for myself … even to take a bath for myself.’ (P38, Male, 37 years)

The following quotes are examples of the participants’ experiences after rehabilitation:

‘It’s very good … because I’m able to do some things … to wash, to go to the shop by myself, not asking somebody.’ (P30, Male, 64 years)

‘Positive … I’m moving better, and I can do the things that I was not able to do before.’ (P28, Female, 42 years)

### Change in activities and participation before and after rehabilitation in children

For children, the activity limitations and participation restriction areas assessed included self-care, play, recreation and leisure, mobility, community, social and civic life, and major life areas related to school/education (Table 5).

**TABLE 5:** Changes in activities and participation before and after rehabilitation for child participants (*N* = 17).

Variable	Before		After		*P*
	*n*	%	*n*	%	
**Self-care (eating, washing, dressing)**					0.011*
No difficulty or independent	1	5.88	2	11.76	
Mild or moderate difficulty (needs some assistance or slow and effortful)	5	29.41	1	52.38	
Severe or complete difficulty (someone else completes most or all of activity)	9	52.94	1	5.88	
Not specified	2	11.76	2	11.76	
**Play, recreation and leisure**					0.476
No difficulty or independent	4	23.52	6	35.29	
Mild or moderate difficulty	4	23.52	6	35.29	
Severe or complete difficulty	1	5.88	-	-	
Not specified	8	52.38	5	29.41	
**Mobility**					0.033*
No difficulty or independent in community	3	17.64	7	41.17	
Mild or moderate difficulty (inside and outside house)	7	41.17	9	52.94	
Severe or complete difficulty (Needs assistance)	5	29.41	0	0.00	
Not specified	2	11.76	2	11.76	
**Community, social, and civic life (socialise, visiting)**					0.019*
No difficulty (independent or often)	3	17.64	10	58.85	
Mild or moderate difficulty (sometimes or with a familiar person only)	9	52.94	5	29.41	
Severe or complete difficulty (limited communication)	3	17.64	0	0.00	
Not specified	2	11.76	2	11.76	
**Major life areas (school or education)**					0.169
No difficulty (mainstream school)	3	17.64	7	41.17	
Mild or moderate difficulty (special school)	3	17.64	1	5.88	
Severe or complete difficulty (full-time care)	1	5.88	0	0.00	
Not at crèche	5	29.41	3	17.64	
Not specified	5	29.41	6	35.29	

Note:

* Significant ≤ 0.050.

Notable changes were observed in several domains that are elaborated in the following subsections.

#### Self-care (eating, washing, dressing)

Three caregivers noticed that their children showed improved independence in self-care such as eating and feeding themselves with some assistance or independently. However, they still require assistance with tasks such as dressing and bathing. While there was a statistically significant improvement (*p* = 0.011) in self-care after rehabilitation, most of the children still need some support from their caregivers as they were young. The following comments are examples of the participants’ experiences:

‘I just take the pyjamas and put it on the bed … he can take off … clothes … and wear … pyjamas.’ (Caregiver, male child, 5 years)

‘I am bathing him … the head and the face. He can bath the body … I wipe him … give him the cloth … to wash himself … every day.’ (Caregiver, female child, 6 years)

Rehabilitation services trained a mother on how to assist her child with feeding, and she said:

‘It’s helping a lot … some of things that I don’t know. They tell me that if you go home you can do like this.’ (Caregiver, male child, 2 years)

#### Play, recreation, and leisure

Just over half of the participants mentioned playing. Most children were able to play, but some limitations were reported in playing with others before rehabilitation. One participant said:

‘She was playing a little bit … but she likes sitting at home.’ (Caregiver, female child, 3 years)

Most children (11.76%) play with others in different settings after rehabilitation, but three children still found it mildly difficult. Only one child has severe to moderate difficulty while playing after rehabilitation and his caregiver described the situation as follows:

‘He seemed to be very angry. Instead of getting a toy and play with a toy, he gets a toy and throw it away.’ (Caregiver, male child, 5 years)

#### Mobility

A statistically significant improvement in mobility was observed (*p* = 0.033), and most children achieved independence in moving within their homes after rehabilitation. Prior to receiving rehabilitation, only 17.64% of children were able to move without difficulty, and 70.58% had mild to severe problems with mobility. The youngest child falling into this category was 21 months old. Two caregivers explained it as follows:

‘He was still improving. He could move on his buttocks. He did it on that time when I was not coming here.’ (Caregiver, male child, 5 years)

‘No balance [*to enable walking*]. She was only sitting.’ (Caregiver, female child, 3 years)

A statistically significant improvement (*p* = 0.033) was reported for mobility in the children after rehabilitation. None of the children were reported to have severe or complete difficulty in mobility after rehabilitation. Even though some children had not reached the developmental milestone of walking, most were walking inside and outside the house. This was expressed as follows:

‘He’s improved … [*he is*] moving around everywhere … the movement started to improve, like kicking. He didn’t know how to use his leg.’ (Caregiver, male child, 2 years)

‘He’s moving around. He can sit … in the chair …. He is able to move. He can get out of the chair and crawl.’ (Caregiver, male child, 4 years)

#### Community, social, and civic life

Social participation was affected for most children (70.58%) because of inability to use language. Many children showed statistically significant improvement (*p* = 0.019) in socialising and communicating with others, enabling them to participate more fully in social activities. Visiting was rarely mentioned, but improvement after therapy was indicated for four children who were now able to communicate and whose behaviour improved, thus allowing them to socialise, as seen in the following comments:

‘He can communicate … can now say words … knows [*his*] sibling’s names … He can hear the song that he loves, he can sing, but not the lyrics.’ (Caregiver, male child, 3 years)

‘He is … comfortable talking to others. He … participates. The behaviour started to change since I attended [*rehabilitation*].’ (Caregiver, male child, 2 years)

#### Major life areas (school and education)

Some children faced difficulties with schooling because of communication difficulties and challenging behaviour, but improvements were observed in these two areas after rehabilitation. Thirty-five per cent of the children were attending or had previously attended a crèche or school before rehabilitation. One child faced severe difficulties with schooling because of communication difficulties and challenging behaviour. His caregiver explained that:

‘He has problems at crèche, behaviour and can’t communicate. It was difficult because … he gets agitated and become angry … maybe he is crying … you grab him, he bites you.’ (Caregiver, male child, 3 years)

Positive change after rehabilitation was noticed for all school-going children. All children at mainstream schools were reported to have no difficulty with learning, and an improvement in communication abilities allowed another to successfully attend school after rehabilitation. This was expressed as follows:

‘After … the help that we got from the rehab, she was able to go back to school and started writing again. She even got an award for being the first one in her grade, you know.’ (Caregiver, female child, 6 years)

‘He is in school now. He is speaking with everybody … so people understand him … he understands them … his schooling days … it’s easier for him.’ (Caregiver, male child, 2 years)

### Feeling about self (self-perception) and quality of life in children

Overall, caregivers reported more positive self-perception and improved QoL in children after receiving rehabilitation services. Statistically significant improvements were observed in both aspects (*p* = 0.008 and *p* = 0.017, respectively), although some caregivers attributed these improvements to their own acceptance and adaptations to the issues rather than changes in function (Table 6).

**TABLE 6:** Change in feeling about self and quality of life before and after rehabilitation perceived by caregivers for child participants (*N* = 21).

Variable	Before		After		*P*
	*n*	%	*n*	%	
**Feeling about self (self-perception)**					
Good	3	17.64	13	35.29	0.008*
Neither good nor bad	6	35.29	2	11.76	
Bad	6	35.29	0	0.00	
Not specified	2	11.76	2	11.76	
**QoL**					
Good	1	5.88	7	41.17	0.017*
Neither good nor bad	7	41.17	8	47.05	
Bad	8	47.05	1	5.88	
Not specified	1	5.88	1	5.88	

Note:

* Significant ≤ 0.050.

QoL, quality of life.

The following comments are examples of the different participants’ perceptions of their child’s experiences before rehabilitation:

‘I don’t know how to put this into perspective … some of his attitude or emotions … it’s only now that we can see it coming up.’ (Caregiver, female child, 3 years)

‘His quality of life … I think … he’s in between … he is going to be fine … it’s difficult for me … his life is bad.’ (Caregiver, male child, 4 years)

The following comments are examples of the participants’ experiences after rehabilitation:

‘So now, she’s a very happy, playful child.’ (Caregiver, female child, 6 years).

‘He is going to have a nice life.’ (Caregiver, male child, 4 years)

## Discussion

The findings of this study provided valuable insights into the outcomes of rehabilitation services provided by an interprofessional team including physiotherapists, occupational therapists and speech and language therapists at the PHC level. The study explored the perceived changes in activity limitations, participation restrictions, well-being and QoL from the perspectives of service users. The results indicated that both adult and caregivers of child service users experienced statistically significant improvements in activity participation-specific domains.

For adult participants, significant improvements were observed in the domains of self-care and mobility. Over half of the adults achieved full independence in self-care, while others still required some assistance, leading to a reduced burden on half of the caregivers. Similarly, with the exception of one adult, all participants reported improved mobility within their homes and yards, with over 40% being able to mobilise in the community. This change was also reported in another study on outcomes of PHC rehabilitation in clients living with human immunodeficiency virus (HIV) in South Africa (Cobbing, Hanass-Hancock & Myezwa 2017). These positive changes reflect the impact of rehabilitation services on individuals’ functional abilities and overall satisfaction with their participation. Similarly, for children, significant improvements were found in self-care and mobility. While only a few children achieved complete independence, the assistance they required became more age-appropriate after receiving rehabilitation, leading to improved participation in daily activities. Notably, communication improvements in children facilitated socialisation within the family and community, and some children were able to attend school successfully.

The positive outcomes reported in this study align with previous research results that demonstrated the positive impact of rehabilitation interventions on functional outcomes and QoL. Global studies report similar improvements in mobility, self-care, and social participation following PHC rehabilitation interventions (Bolt et al. 2019b; De Coninck et al. 2017; Eklund et al. 2015; Kjerstad & Tuntland 2016; Meisingset et al. 2021). These findings support the WHO’s (2019) emphasis on the provision of rehabilitation services at the PHC level for individuals of all ages.

However, it is essential to acknowledge that some domains, such as domestic, community, social, and civic life; major life areas related to work for adult participants; play, recreation, and leisure; and school/education for child participants did not show statistically significant positive changes. Activity limitations in these domains are not easily addressed in the clinic environment. A possible explanation is that providing rehabilitation requires a more comprehensive approach and consideration of the specific environment in which the activities occur. Addressing these activity limitations may involve incorporating service delivery models that move services out of the CHCs, such as outreach through home visits and task shifting to community rehabilitation workers, to better integrate rehabilitation at the PHC level (Larsson-Lund & Nyman 2017).

The study also reflected the perceptions of the users to realistically evaluate the outcomes of rehabilitation interventions. When the perceptions of service users are probed, it is important to consider a person-centred approach that is user-friendly and not influenced by power-dynamics. Tailoring assessment tools to the specific population being assessed is crucial to obtain accurate and relevant information (Dronavalli & Thompson 2015). In this study, simple scales and structured interviews allowed participants to express their views on changes in activities and participation and considered caregivers’ feelings and QoL in relation to caring for their child and the impact of rehabilitation on them (Irwin et al. 2012).

Although the findings in this study showed that rehabilitation intervention has a positive effect, the authors are aware that the participants are the cohort who utilised rehabilitation services and that assessment and comparison with participants who did not engage in rehabilitation would be more reflective of the effect of rehabilitation. The sustainability of the positive rehabilitation outcomes is an important factor to consider, especially at PHC level. Outcomes can be enhanced by including other models, providing support for caregivers of children with disabilities, and creating community-based groups for adults with neuromusculoskeletal impairments (Scheffler & Mash 2019).

### Limitations of the study

The study sample was not heterogeneous in terms of disability because the participants presented predominantly with neuromusculoskeletal impairments, which may not fully represent the broader range of rehabilitation needs in the district. Only participants who were able to access the clinics for rehabilitation over three sessions were included, and thus, the results cannot be generalised to those who had limited or no access to these services. The measure of QoL in children was more of a representation of the caregivers’ perceptions than of the children themselves.

## Conclusion

The interprofessional PHC rehabilitation services showed positive improvements in activities and participation, self-perception, and QoL from the perspectives of service users. This study provides valuable insights into the outcomes of PHC rehabilitation services and emphasises the importance of considering person-centred approaches and service-user perspectives when evaluating rehabilitation interventions.

The following recommendations are proposed for future research and policy implementation:

- Adapting services to varying environments: While clinic-based services or the clinic model proved effective in certain domains, it is important to explore alternative service delivery models beyond the clinic setting. For instance, home visits and task shifting should be considered to address domains such as domestic life, which may be better attended to in patients’ homes.
- Addressing gaps in service provision: Community-based support groups and self-help groups should be incorporated into interventions to address social activities and work-related challenges for adults. The self-help groups should explore self-employment opportunities in contexts with limited employment opportunities.
- Exploring barriers to access: Future research should focus on understanding the barriers that prevent individuals with disabilities from accessing rehabilitation services, particularly those who require rehabilitation but are not accessing these services. Identifying these barriers will enable targeted strategies to improve access and inclusivity in rehabilitation service delivery.
- Patient evaluation of service: Longitudinal studies should be conducted to assess the sustainability of positive rehabilitation outcomes over an extended period. Long-term evaluations will provide valuable insights into the lasting impact of PHC-level rehabilitation interventions and guide efforts to integrate rehabilitation services into PHC.
- Strengthen service-user participation and involvement: The voice of the service user is important and must be incorporated when evaluating the effectiveness of PHC rehabilitation in activity limitations, participation restrictions, and service planning. Such evaluation measures should consider not only the functional abilities of service users but also the impact of rehabilitation on their caregivers and their QoL.
- Person-centred measures: Develop and implement person-centred evaluation tools tailored to the specific population being assessed. Service providers should routinely report on outcomes related to body functioning, activities, and participation based on standardised tools for each patient to support the effectiveness of rehabilitation.
- Advocacy for education and employment opportunities: Collaborative strategies between the health, disability, and education sectors to strengthen advocacy efforts are necessary to ensure children have access to inclusive and quality education in alignment with the Sustainable Development Goals.

By implementing these recommendations, rehabilitation services can become more effective, inclusive, and responsive to the needs of service users. Furthermore, rehabilitation service providers can work collaboratively to influence policy through tangible outputs to demonstrate the impact of rehabilitation interventions and promote the overall well-being and social inclusion of individuals with disabilities. These measures can achieve recognition of rehabilitation services and effective integration of these services into PHC.
